# The Impact of Micro/Nanoplastics on Human Hematopoietic Function: Exposure, Deposition, Toxicity, and Mitigation Strategies

**DOI:** 10.3390/toxics14040328

**Published:** 2026-04-15

**Authors:** Yao Zhou, Xuanwei Chen, Jin Chen, Jian Xu

**Affiliations:** 1 School of Public Health, Zhejiang Chinese Medical University, Hangzhou 310053, China; 202112213602021@zcmu.edu.cn; 2 School of Medical Technology and Information Engineering, Zhejiang Chinese Medical University, Hangzhou 310053, China; chenjin0425@zcmu.edu.cn

**Keywords:** micro- and nanoplastics, human body accumulation, hematopoietic toxicity

## Abstract

The continuous accumulation of micro- and nanoplastics in the human living environment and their deposition in various organs of the body have become a global public health concern with the widespread use of plastic products. This review summarizes the main categories of micro- and nanoplastics entering the body through dietary intake and air inhalation, based on human exposure pathways. By integrating existing literature data, this review estimates the daily intake and excretion of micro- and nanoplastics in humans, summarizes evidence regarding their potential deposition patterns in blood cells and hematopoietic-related organs, mainly inferred from animal and in vitro studies, and discusses the possible impacts of such deposition on hematopoietic function. Furthermore, the toxic effects and potential hazards of micro- and nanoplastics on the human hematopoietic system at both cellular and animal levels, along with the underlying molecular mechanisms, are comprehensively reviewed. From the dual perspectives of environmental governance and bodily protection, exploratory research ideas are proposed, including biodegradation strategies and the application of medicinal and edible homologous substances. This review aims to provide insights for reducing the risk of hematopoietic system diseases and preventing harm caused by micro- and nanoplastics to the human body in the future.

## 1. Introduction

According to statistics, global plastic production reached 413.8 million tonnes in 2023, with output projected to double over the next two decades [1]. However, only 6% of these plastics were recycled, while up to 94% were estimated to be landfilled or released into the environment via various pathways [2]. Subsequently, this plastic waste is transformed into various harmful substances through a variety of complex environmental processes, specifically microplastics (MPs) and nanoplastics (NPs), through multiple mechanisms.

**Figure 1:**
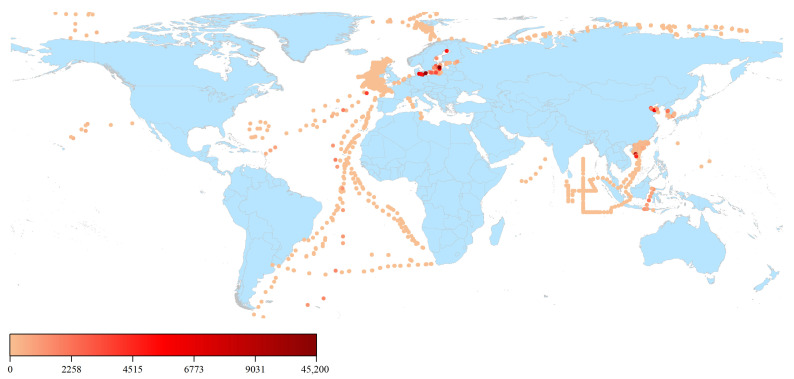
Detection of submerged microplastics (MPs) in the global marine environment (plotted with Origin; data sourced from Zhao et al. [4]). Note: The distribution of MPs in the global marine environment is represented by dots; lighter colors indicate lower microplastic concentrations, whereas darker colors denote higher concentrations. Unit particles/m^3^.

Driven by human activities, micro- and nanoplastics (MNPs) are widely distributed in the natural environment, causing serious pollution. Their presence has been documented across the global marine environment, spanning from coastlines to polar seas and from surface waters to the Mariana Trench (Figure 1). Furthermore, terrestrial ecosystems are considered to contain 4 to 23 times higher levels of MNPs compared to marine environments, with concentrations ranging from 300 to 67,500 mg/kg [3,4]. Due to their pervasive presence, human exposure to MNPs is inevitable. Indeed, the latest study by Stefan et al. (2024) reported the detection of MNP particles in human biological samples, such as feces, lung tissue, blood, breast milk, and placenta, confirming the hypothesis that environmental MNPs accumulate in the human body [5].

To date, the health risk factors associated with exposure to MNPs have not been fully clarified, and research on the effects of MNPs on the hematopoietic system is particularly lacking. In this narrative review, we synthesize existing literature in a descriptive manner, aiming to investigate the deposition patterns and possible toxicological mechanisms of MNPs in the human hematopoietic system, explore their potential effects on hematopoietic function, and explore approaches to address MNP-related issues, such as biodegradation and the application of medicinal and edible homologous substances, thereby providing research ideas for future prevention and control measures.

## 2. Methodology

Searches were performed across the PubMed and Scopus databases, restricted to articles published between 2000 and 2025, with an emphasis on the most up-to-date literature. Priority was given to studies investigating human populations or experiments conducted using human-derived cells. Clinical studies, original research, narrative reviews, and meta-analyses were retrieved. Titles, abstracts, and full texts were screened based on relevance to MNPs and the hematopoietic system. Keywords searched included various combinations of the following: “microplastics”, “nanoplastics”, “micro/nanoplastics”, “human exposure”, “accumulation”, “daily accumulation”, “hematopoietic function”, “hematopoietic organs”, “blood cells”, “leukocytes”, “erythrocytes”, “thrombocytes”, “bone marrow”, “yolk sac”, “liver”, “spleen”, “kidney”, “deposition pattern”, “deposition toxicity”, “prenatal hematopoiesis”, “postnatal hematopoiesis”, “toxicity”, “cytotoxicity”, “immunotoxicity”, “inflammatory response”, “metabolic disorder”, “toxicological mechanisms”, “environmental reduction”, “toxicity mitigation”, “gut microbiota”, “medicinal and edible homology”. In addition, the references of many of the articles identified by the above search strategy were reviewed, and foundational references outside the search window were included where necessary for context.

### Inclusion/Exclusion Criteria and Limitations

Studies were included in our narrative review if they (1) described sources or daily accumulation of MNPs in human populations, (2) reported deposition patterns in blood cells or hematopoietic-related organs (liver, spleen, bone marrow, yolk sac, etc.), (3) addressed potential effects on hematopoietic function before or after birth, (4) explored toxicological mechanisms at the cellular or organ level (e.g., inflammatory response, metabolic disorders, immunotoxicity), or (5) proposed mitigation strategies from environmental reduction or “medicinal and edible homology” perspectives.

Studies were excluded if they (1) were not indexed in PubMed or Scopus, (2) were published earlier than 2000, (3) were not written in English, (4) investigated non-plastic particles without specific relevance to plastics, (5) were conference abstracts, commentaries, or editorials without original data, or (6) were duplicate publications. Studies included in this review were available in full text online.

Limitations of this study design include (1) without including other more comprehensive databases, (2) the fact that it is a narrative review and not systematic, and therefore (3) the absence of formal quality assessment or meta-analysis.

## 3. Sources and Daily Accumulation Amount Estimation of Micro- and Nanoplastics in Human Populations

Plastics in the environment are primarily broken down through oxidation, hydrolysis, photodegradation, mechanical degradation, and biodegradation, generating fragments of MPs and NPs in various forms and sizes. These MNPs subsequently enter the human body via inhalation and ingestion. Consequently, airborne and dietary MNPs are identified as the primary vectors affecting human health [6,7,8] (Table 1). In academia, MPs intentionally manufactured and added to commercial products are typically termed primary MPs, whereas those resulting from the degradation of larger plastic materials are referred to as secondary MPs. However, NPs are generally formed from the fragmentation of MPs and are defined as particles with a size less than 1000 nm. It is estimated that primary MNPs account for only 15–30% of those released into the natural environment, with the remainder being secondary [9].

Exposure to MNPs may vary among populations due to environmental, occupational, and lifestyle factors. Early life stages are generally more vulnerable to environmental stressors because of their immature organ systems and developing physiological barriers; however, the specific health consequences of MNP exposure in infants remain incompletely understood. Therefore, understanding the accumulation of MNPs in infants is of paramount importance. Zuri et al. (2023) used keywords such as “MP in outdoor and indoor air, indoor dust, food including beverages and water and human intake” to filter 115 publications from Scopus and PubMed [10]. Based on the data presented in these publications, they calculated the daily amount of MNPs entering the human body. We adopted the statistical method of Zuri et al. to analyze the accumulation of MNPs in both infants and adults [10]. To assess the population-level exposure to MNPs, we calculated the Total Daily Intake (TDI), which comprised the Inhaled Dose (ID) of MPs from air, the Dust Ingested (DI) and the Estimated Daily Intake (EDI) from dietary sources. As shown in Table 2, all intake values were standardized by calculating them per unit of average bodyweight (bw) for better comparability. The results indicate a TDI of 193 particles/kg bw for infants, and 210 particles/kg bw for adults, revealing that the TDI for infants is similar to that for adults, among which the ID is nearly twice that of adults [10]. However, given that MNPs undergo metabolic processes in the human body similar to food, the fraction that truly exerts an effect is their accumulated amount. By reviewing studies on MNPs in human excreta, this paper utilized the MNPs content in feces as the Daily Output (DO) to estimate daily MNPs excretion.

To estimate the daily accumulation of MPs in the human body, we adopted the parameter settings for adults from Zuri et al. and set the average adult body weight at 70 kg [10]. On this basis, with reference to the Child Growth Standards published by the World Health Organization (WHO) in 2006, we set the average body weight of 6-month-old infants at 8 kg. Based on this, daily total fecal weight in healthy individuals was further obtained through a literature survey: 4–415 g for adults and 6.7–44.7 g for 6-month-old infants [11,12]. The maximum daily total fecal weight was used in the calculation of daily accumulation. This approach assumes that, within the normal physiological range, the body excretes MPs to the greatest extent possible, thereby allowing us to assess whether MPs can still accumulate in the human body.

However, even under this conservative assumption of maximized excretion, the calculated results remain alarming: 198 particles/kg bw for adults and 44 particles/kg bw for infants. Notably, although data indicate that the tolerable daily intake (TDI) of MNPs for infants is similar to that for adults, their daily accumulated dose is significantly lower. From a metabolic rate perspective, although infants ingest relatively high quantities of MPs, the majority may be excreted through faster metabolic processes, potentially resulting in a relatively limited health risk [13,14].

**Figure 2:**
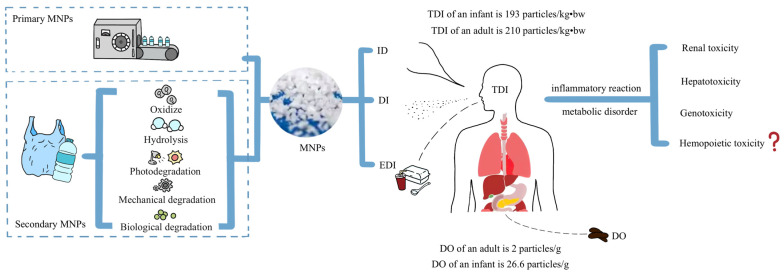
Environmental sources, human absorption, and toxicity effects of micro- and nanoplastics (MNPs). Note: From left to right, the sources of MNPs, pathways of human exposure, the reported amounts of intake and excretion in specific populations, and the resulting toxicity are presented (Renal toxicity [15,16,17,18,19,20]; Hepatotoxicity [21,22,23,24,25,26,27,28]; Genotoxicity [29,30,31,32,33,34,35]). TDI: Total Daily Intake; ID: Inhaled Dose; DI: Dust Ingested; EDI: Estimated Daily Intake; DO: Daily Output.

It should be noted that total daily fecal weight is significantly affected by various factors such as dietary habits and individual differences. To estimate the daily accumulation level of MPs in the human body as conservatively as possible, this study comprehensively referred to literature data and practical experience when setting parameters, and selected the maximum values within the healthy range for calculation. It is worth clarifying that the various settings in the model may vary with age, lifestyle, and health status. Therefore, the obtained results can only serve as approximate values for the daily accumulation of MPs. How long the MNPs ingested on a given day will continue to be excreted, and the amount excreted each day, remains a topic that requires further investigation. Nevertheless, this result still offers some reference value for quantifying exposure levels in both infants and adults. As plastic pollution intensifies globally, the issue of MNPs accumulation in humans may become increasingly severe, warranting serious attention (Figure 2).

**Table 2:** Predicted estimate of daily MNPs accumulation in humans.

Category	Assumed Body Weight (kg)	TDI [10] (Particles/kg bw)				DO [13,14] (Particles/g)	Daily Total Fecal Weight [11,12] (g)	Daily Accumulation Amount ^1^ (Particles/kg bw)
		ID	DI	EDI	Total			
Adult	70	114	0.21	96	210	2	4–415	198
Infant (around 6 months of age)	8	190	3.4	NC ^2^	193	26.6	6.7–44.7	44

^1^ Calculated at the maximum daily total fecal weight. ^2^ NC = Not calculated due to the lack of information on MP concentration in infant food formulas. TDI: Total Daily Intake; ID: Inhaled Dose; DI: Dust Ingested; EDI: Estimated Daily Intake; DO: Daily Output.

## 4. Deposition Patterns of Micro- and Nanoplastics in Blood Cells and Hematopoietic-Related Organs and Their Potential Impact on Human Hematopoietic Function

### 4.1. Deposition Patterns in Blood Cells

**Figure 3:**
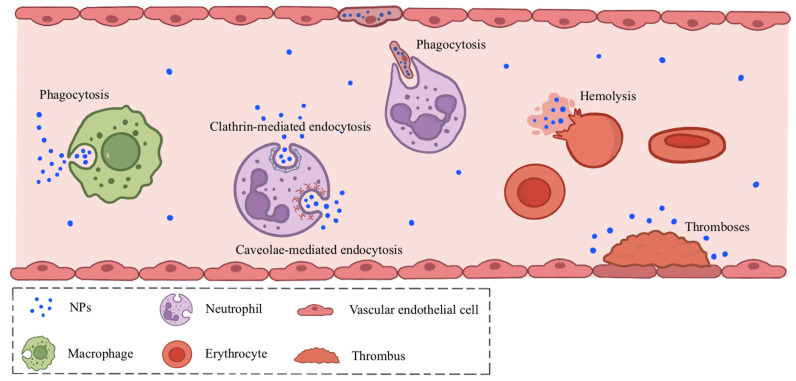
Deposition of nanoplastics (NPs) in Blood Cells.

Recent studies indicate that ingestion of NPs induces alterations in peripheral blood cell composition (Figure 3).

Within leukocytes, NPs are prone to deposit in neutrophils and macrophages. As the most abundant leukocyte population, the accumulation of NPs within neutrophils significantly impacts human immune function. In vitro studies using giant unilamellar vesicles (GUVs), small unilamellar vesicles (SUVs), and giant plasma membrane vesicles (GPMVs) have shown that NPs enter neutrophils via interactions with the cell membrane and endocytosis mediated by clathrin and caveolin [36]. Beyond direct phagocytosis, another common pathway that involves NPs is indirect uptake via phagocytosis of endothelial cells contaminated with NPs. An in vitro experiment using immortalized human cerebral microvascular endothelial cells (hCMEC/D3) showed that when NPs enter endothelial cells, they will trigger the production of reactive oxygen species (ROS), activate nuclear factor kappa-B (NF-κB), and induce tumor necrosis factor synthesis, ultimately causing necrotic apoptosis in endothelial cells [37]. Neutrophils then indirectly accumulate NPs within themselves by phagocytosing these damaged endothelial cells [38]. In contrast, macrophages primarily achieve the accumulation of MP particles within leukocytes through direct phagocytosis [36].

Currently, no studies have directly elucidated the deposition patterns of NPs within erythrocytes, and research into the effects of MNPs on erythrocytes remains in its infancy. In an in vitro study on human erythrocytes, Hwang et al. (2020) observed that direct exposure to MNPs of a certain size and concentration induced hemolysis [39]. The study further revealed that polystyrene (PS) particles smaller than the average diameter of erythrocytes (6–8 μm) exhibited stronger cytotoxicity across all tested concentrations due to their larger surface area, whereas larger PS particles (diameters of 10–100 μm) showed no hemolytic effects. They attributed this primarily to weak interactions, such as van der Waals forces, which enhanced the adhesion of smaller particles to erythrocytes [39]. Based on existing literature and postulated mechanisms, we further speculate that, on the one hand, the entry of NPs into cells may occur via osmosis or active endocytosis, requiring specific channel proteins such as clathrin and caveolin for mediation; however, as mature erythrocytes lack these proteins, most NPs cannot enter except for a small fraction via osmosis [36]. On the other hand, the inherent fragility of erythrocytes means that once NPs enter or simply come into contact with the cells, they may readily cause cellular disruption leading to hemolysis, after which they are released again. Consequently, accumulation in erythrocytes is unlikely [39,40,41].

Compared with that in erythrocytes, the deposition of NPs in thrombocytes seems more plausible, given that existing studies have confirmed the presence of NPs in human thrombi [42]. Using in vitro experiments with human blood and thrombocytes, McGuinnes et al. found that NPs of identical particle size can induce platelet activation through different pathways depending on their surface charge [43]. Cationic NPs induce platelet activation and aggregation by disrupting the cell membrane, whereas anionic NPs promote aggregation via the classical pathway. Concurrently, an inflammatory response is triggered through the JAK1/STAT3/TF signaling pathway, as demonstrated by combined in vivo mouse studies and in vitro experiments, leading to coagulation activation and thrombus formation, where NPs subsequently deposit. This process may contribute to cardiovascular damage [44].

### 4.2. Deposition Patterns of Micro- and Nanoplastics in Hematopoietic-Related Organs of Animals

After being absorbed in the gastrointestinal tract, MNPs can cross the intestinal barrier and enter the systemic circulation, reaching various organs throughout the body, with primary deposition and distribution occurring in organs such as the liver and kidneys [45,46]. Multiple studies have shown that the distribution of MNPs in animal organs is related to their particle size and surface modifications. Within a certain size range, smaller MNPs are more likely to accumulate in tissues, with finer particles tending to deposit in organs like the liver, spleen, and kidneys, while larger particles are more prone to accumulate in the gastrointestinal tract [47]. However, nanoparticles smaller than 10 nm can be rapidly filtered by the glomeruli and excreted in urine [48]. Deng et al. investigated the distribution of MPs with sizes of 20 μm and 5 μm in vivo and found that both types accumulated in the liver and kidneys of mice [49]. Notably, the accumulation of 5 μm MPs in the kidneys was significantly higher than that of 20 μm MPs, while the opposite pattern was observed in the liver. This may be because the liver, via the enterohepatic axis, is more readily exposed to MPs ingested through the intestine, leading to initial deposition of larger particles in the liver. In contrast, smaller MPs are more likely to accumulate extensively in the kidneys due to filtration and reabsorption processes. As the central regulator of the hematopoietic system, the kidney plays an indispensable role in hematopoiesis both before and after birth. However, since the liver normally functions hematopoietically only during the prenatal period, the impact of MNP deposition on its hematopoietic role should primarily be considered in the context of the prenatal phase. Studies have detected PS-NPs in the liver, lungs, and kidneys of fetuses following acute maternal pulmonary exposure to PS-NPs during late pregnancy in rats. This suggests that MNPs may cross the maternal placental barrier, deposit in fetal liver and kidneys, and thereby exert potential effects on fetal hematopoietic function [50,51].

Furthermore, research by Walczak et al. indicated that the distribution of NPs in animal organs was related to their surface charge [52]. They investigated the bioavailability and deposition patterns of NPs with different charges following a single oral administration in rats. Fluorescence quantification and organ imaging revealed that animal organs absorbed negatively charged NPs more readily than other types, and these negatively charged NPs were detected in almost all examined organs.

However, as these findings are derived from rodent models using particle doses, types, and administration routes that differ substantially from real-world human exposure, caution is warranted in extrapolating these organ distribution patterns to humans, and direct evidence of MNP deposition in human hematopoietic organs at environmentally relevant concentrations remains lacking.

### 4.3. Potential Effects of Micro- and Nanoplastics on Human Hematopoietic Function

A growing body of evidence has demonstrated that MNPs can accumulate in the human body. MPs have been detected in human bone marrow at a concentration of 51.29 μg/g and in human blood at 1.6 μg/mL [53,54]. However, due to ethical constraints, clinical studies investigating the effects of MNPs exposure in humans remain lacking, and reports from in vitro experiments using human blood cells as well as population-based studies are still scarce. Guo et al. found that in colony-forming assays, exposure to 0.1 mg/mL of PS-NPs for 10 days significantly inhibited the growth of colony-forming unit–granulocyte-monocyte (CFU-GM), colony-forming unit-erythrocyte (CFU-E), and burst-forming unit-erythroid (BFU-E) colonies derived from human CD34+ hematopoietic stem/progenitor cells (HSPCs) [55]. This result indicated that PS-NP exposure suppressed the self-renewal, unlimited proliferative and differentiation capacity of HSPCs, thereby impairing human hematopoietic function. Beyond this, the literature directly investigating the impact of NPs on human hematopoietic function is extremely limited.

Given that the accumulation of MNPs in the human body appears to be a partially irreversible process, and that the overall trend is towards an increase with age. Therefore, the accumulation level of MNPs in the human body may be further elevated. In this context, although relatively high doses of MNPs are adopted in current cellular and animal studies, the related findings can still provide important references for the evaluation of potential damage induced by MNPs to the human hematopoietic system. Moreover, these results are helpful for the exploration of the underlying toxicological mechanisms. Accordingly, combined with the existing results of relevant animal studies, the potential effects of MNPs on human hematopoietic function and the corresponding toxicological mechanisms are preliminarily analyzed and speculated in this study.

#### 4.3.1. Potential Effects of Micro- and Nanoplastics on Hematopoietic Function Before Birth

Organs involved in prenatal hematopoiesis in humans include the yolk sac, liver, spleen, and lymph nodes. Furthermore, the functional role of the kidneys as a regulatory organ in hematopoiesis warrants significant attention. Therefore, the impact of MNPs on the functions associated with these organs is analyzed.

Regarding the yolk sac, studies investigating PS-NPs-induced developmental toxicity and vascular toxicity in zebrafish have found that PS-NPs can induce vascular toxicity and yolk sac edema in the yolk sac region of transgenic zebrafish [56]. Zebrafish serve as a classic animal model for studying embryonic development, and findings from such models offer valuable reference insights for human embryos. The liver during the embryonic period is also a crucial hematopoietic organ. In vitro studies revealed that liver organoids derived from human pluripotent stem cells (hPSCs), when exposed to 1 μm PS-MPs at a concentration of 0.25 μg/mL, exhibited increased cytotoxicity, including elevated apoptotic cell counts, alongside heightened lipotoxicity. Furthermore, PS-MPs led to lipid accumulation, reduced ATP generation, increased ROS production, and the release of inflammatory markers interleukin (IL)-6 and COL1A1. These alterations elevate the risk of hepatic steatosis and fibrosis [9]. Additionally, following MP uptake, human embryonic kidney cells are also affected, showing morphological changes, reduced viability, increased intracellular ROS, decreased glycolytic activity, and downregulated expression of ROS-scavenging marker genes such as GAPDH, SOD, and CAT. These effects contribute to disturbances in energy and lipid metabolism [9,57]. We summarized the above content in Table 3.

Admittedly, the findings discussed above are primarily derived from animal studies and in vitro experiments and use exposure concentrations that may substantially exceed those encountered in vivo. Nevertheless, it is worth noting that the cells involved in the analyses of both the liver and kidney are of human origin. To some extent, this may enhance the translational relevance of the results, as human-derived cell lines are generally considered to better reflect certain physiological characteristics of human tissues. Therefore, while caution is warranted when extrapolating these findings to human conditions, the use of human-derived cells in these studies may offer a degree of supportive evidence for the speculated mechanisms.

#### 4.3.2. Potential Effects of Micro- and Nanoplastics on Hematopoietic Function After Birth

As the primary hematopoietic organ after birth, the bone marrow serves as a critical focal point for analyzing the impact of MNPs on hematopoietic function. In vitro studies have shown that MNPs can impair the migratory capacity of murine pre-osteoblasts and enhance the cellular generation of pre-osteoclasts. Furthermore, they inhibit osteoblast differentiation through inflammatory responses while promoting the transformation of pre-osteoclasts. Additionally, research suggests that MNPs affect the growth and function of osteoblasts and osteocytes via ROS, thereby promoting cellular apoptosis [83]. Although these in vitro findings provide plausible biological mechanisms, their translational relevance is limited by the use of isolated cell cultures under non-physiological exposure conditions, including high concentrations and simplified particle characteristics that do not reflect the complexity of in vivo exposure. Moreover, the extent to which such effects occur at environmentally relevant doses in humans remains unsubstantiated. Further research is needed to determine whether these mechanisms translate to similar effects in humans.

MNPs may also indirectly affect bone marrow hematopoietic function through the peripheral blood. Sun et al. found that after exposure to 0.5 mg of 5 μm PS-MPs for 28 days, peripheral blood leukocyte counts significantly decreased and platelet counts increased in 5-week-old male C57BL/6 mice [84]. Furthermore, MNPs can inhibit the growth of CFU-GM, suggesting that MNP exposure may reduce peripheral blood cell counts by suppressing the differentiation of bone marrow hematopoietic stem cells into granulocytes and megakaryocytes [83,84]. Research by Jing et al. yielded similar results [85]. After 4-week-old male C57BL/6J mice were exposed to 60 μg PS-MNPs of sizes 10 μm, 5 μm, and 80 nm for 42 days, significant reductions were observed in BFU-E, CFU-GM, and colony-forming unit–granulocyte, erythrocyte, monocyte, macrophage (CFU-GEMM). This indicates that PS-MNPs affect the proliferation and differentiation of hematopoietic stem cells (HSCs), impair erythropoiesis, and hinder the generation of mature blood cells. The experiments also revealed that smaller MNPs are more likely to penetrate the circulatory system, damage bone marrow hematopoietic function, and consequently affect changes in peripheral blood cell counts. However, all the above experiments were conducted on animals. The doses administered (e.g., 0.5 mg or 60 μg over 28–42 days) differ by orders of magnitude from the chronic, low-level environmental exposures experienced by humans. Moreover, the particle types and exposure routes utilized in these preclinical models do not fully replicate the complexity of real-world human contact with MNPs. Consequently, these findings serve solely as cautionary notes that raise legitimate concerns about potential human hazard; they do not permit direct extrapolation to definitive human health outcomes.

**Figure 4:**
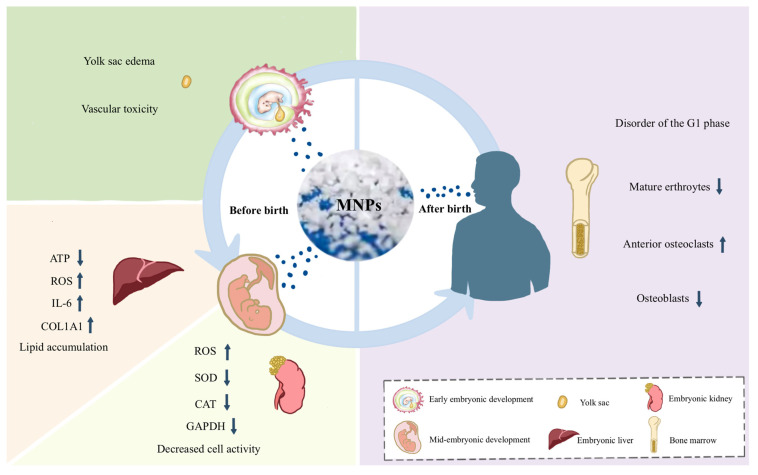
Potential impact of micro- and nanoplastics (MNPs) exposure on hematopoietic-related organs across different human life stages. Note: The left side represents the period before birth, with the upper part depicting the potential effects of MNPs on the yolk sac during early embryonic development, and the lower part depicting the potential effects of MNPs on the liver and kidneys during mid-embryonic development. The right side represents the period after birth, depicting the potential effects of MNPs on the bone marrow. “↑” indicates an increase in the level of expressed of this indicator; while “↓” indicates a decrease in the level of expression of this indicator. ATP: Adenosine Triphosphate; ROS: Reactive Oxygen Species; IL-6: Interleukin-6; COL1A1: Collagen Type I Alpha 1 Chain; SOD: Superoxide Dismutase; CAT: Catalase; GAPDH: Glyceraldehyde-3-phosphate Dehydrogenase.

With the advancement of detection technologies, transcriptomics has opened new avenues for molecular mechanism research from a genome-wide macro perspective. The JAK/STAT pathway, an evolutionarily conserved signaling cascade in eukaryotes, regulates numerous critical biological processes such as cell proliferation, differentiation, apoptosis, and immune regulation. It plays a pivotal role in various hematological disorders and cancers. In a mouse model exposed to 0.1 mg of 5 µm PS-MPs, Sun et al., through transcriptomic analysis, speculated that MNPs may disrupt hematopoietic stem cell division and lead to hematopoietic dysfunction by modulating the JAK/STAT signaling pathway and affecting the G1/S phase of the cell cycle [84]. These transcriptomic findings preliminarily reveal the core molecular pathways underlying MNP-induced hematopoietic toxicity in animal models, and also provide valuable clues and directional references for further exploring the potential mechanisms of MNP-related hematopoietic impairment in humans (Figure 4).

## 5. Possible Toxicological Mechanisms of Micro- and Nanoplastics Deposition in the Body

While evidence regarding the accumulation of MNPs in the human body can be partially derived from analyses of human blood, tissues, or excretory samples, investigations into the underlying toxicological mechanisms remain largely confined to animal models due to ethical constraints and current technical limitations. Therefore, the mechanisms discussed in this section represent possible toxicological mechanisms in humans, inferred primarily from in vivo animal studies. Nevertheless, some of these findings have been corroborated by in vitro experiments using human-derived cell lines, thereby providing more robust support for our hypotheses.

### 5.1. Mechanisms of Micro- and Nanoplastics Deposition Toxicity at the Cellular Level

#### 5.1.1. Inflammatory Response

Current research widely suggests that exposure to NPs primarily triggers inflammatory responses by activating immune cells and stimulating their secretion of pro-inflammatory cytokines. Researchers have conducted in-depth investigations into the specific toxicological mechanisms of NPs by constructing various experimental models. When the human gastric adenocarcinoma cell line (AGS) was exposed to NPs (44 nm) at a concentration of 10 μg/mL for 1 h, the gene expression of pro-inflammatory cytokines such as IL-6, IL-8, and IL-1β was upregulated, demonstrating a strong activating effect [86]. The Brown team noted in their study that rats exposed to 1 mg NPs (64 nm) for 24 h via inhalation exhibited significant inflammatory responses in their lungs [87]. Concurrently, other studies have found that after seven days of exposure to NPs, both 5 μm PS-MPs and 70 nm nanoparticles accumulated in the gills, liver, and intestines of zebrafish. This accumulation led to hepatocyte necrosis and lipid deposition, with liver tissues displaying typical inflammatory damage such as vacuolization and neutrophil infiltration [86].

#### 5.1.2. Metabolic Disorders

Researchers have also employed metabolomics to analyze the toxicological mechanisms of MNPs from a metabolic perspective. The results indicate that after exposure to NPs (80 nm) for 48 h, the most significantly affected metabolic pathways in human normal hepatocytes (HL-7702/L-02) and human normal lung epithelial cells (BEAS-2B) include the tricarboxylic acid (TCA) cycle and glutathione (GSH) metabolism, both of which are related to mitochondrial function. However, different cell types exhibit varied responses to mitochondrial reactive oxygen species (mROS) generation following NP exposure [88]. Among these, the metabolic function of L-02 cells is more sensitive to exposure at low concentrations of NPs [89]. At 0.0125 mg/mL, L-02 cells exhibited significantly elevated mitochondrial ROS levels in a dose-dependent manner, obvious mitochondrial membrane potential depolarization, and remarkable reductions in basal respiration, maximal respiration, and mitochondrial ATP production. Nontarget metabolomics further demonstrated that the number of differential metabolites in L-02 cells was far higher than that in BEAS-2B cells at this low concentration, accompanied by severe perturbations in mitochondrial-related pathways including the TCA cycle, glutathione metabolism, and purine metabolism. In contrast, BEAS-2B cells showed only a slight increase in mitochondrial ROS, no significant change in mitochondrial membrane potential, and unaltered mitochondrial respiration and ATP production under the same low-concentration exposure, with much slighter metabolic disturbances [90].

Concurrently, under NP exposure, Kupffer cells, specialized macrophages in the liver, oxidize free fatty acids, thereby disrupting hepatic lipid metabolism and increasing the risk of developing metabolic dysfunction-associated fatty liver disease (MAFLD) [38,76].

#### 5.1.3. Immunotoxicity

NPs also exhibit immunotoxicity through immunosuppression and interference with autoimmunity.

In vitro neutrophil functional assays demonstrate that phagocytosis of 7.3 µm NPs can lead to the death of 50–97% of neutrophils within 45 min, while phagocytosis of 49–880 nm NPs can cause rapid death in 49–53% of neutrophils. Furthermore, the ingested NPs can be re-released from dead cells and proceed to damage other neutrophils, thereby inducing immunosuppression [91]. Further research has revealed that 50 nm NPs at 10 μg/mL can be internalized by mouse spleen cells after 3 h of exposure, inhibiting cellular activity by blocking the cell cycle and upregulating the expression of inflammation-related genes, consequently leading to increased apoptosis [92]. Given that T cells constitute the major component of splenocytes, internalized NPs can inhibit the differentiation of T cells into CD8+ T cell subsets by suppressing the PKCθ/NFκB and IL-2R/STAT5 signaling pathways. This process impedes the development and function of cytotoxic T lymphocytes, compromises autoimmune regulation, and elevates the risk of pathogenic infections and cancer [92,93].

In terms of autoimmunity, exposure to NPs induces significant alterations in immune cells at transcriptional levels, including enzyme activity. In an in vitro study, Han et al. found that exposure to 1000 μg/mL of polyvinyl chloride (PVC) and acrylonitrile butadiene styrene (ABS) (sizes: 25–75 μm and 75–200 μm) for 4–5 days triggered an immune response in human peripheral blood mononuclear cells (PBMCs), increasing the release of IL-6 and TNF-α [94]. Other types of MP may also induce the production of pro-inflammatory cytokines such as IL-6, TNF-α, and histamine in a size- and concentration-dependent manner, leading to local autoimmune reactions [9].

This section focuses on the cellular-level toxicity mechanisms of MNPs. The effects described herein are derived exclusively from in vitro studies, and the exposure doses employed may substantially exceed those encountered by humans under real-world conditions. At present, dose–response relationships at environmentally relevant concentrations remain poorly characterized. Notably, to enhance the translational relevance of the findings to the extent possible, each subsection of this section has referenced studies that employed human-derived cell lines, thereby providing as reliable a reference as possible for understanding potential response mechanisms in the human body.

### 5.2. Possible Toxicological Mechanisms of Micro- and Nanoplastics Deposited in Hematopoietic-Related Organs

Existing research indicates that MNPs predominantly exert toxic effects on mammalian hematopoietic-related organs through mechanisms such as metabolic disruption and inflammatory responses. This process involves the activation or inhibition of a series of signaling pathways, ultimately leading to alterations in organ function (Table 4). For instance, a chronic exposure study of PS-NPs in rats found that PS-NPs inhibited the expression of the renal Nrf2 pathway and its downstream genes and proteins, leading to increased malondialdehyde (MDA) levels, reduced antioxidant enzyme activity, and upregulation of apoptosis-related genes, such as Cytc and CASP3. These changes were accompanied by a renal inflammatory response characterized by elevated Cyclooxygenase-2 (COX-2) expression [95]. In a separate study, exposure to PS-MPs was shown to not only inhibit the renal Nrf2 pathway but also activate the ferroptosis/mitochondria-related pathway and ferritinophagy, thereby inducing kidney injury [96]. Morphologically, these molecular disruptions consistently translate into pathological damage, a finding supported by multiple studies that have identified lymphocyte infiltration, mild glomerular atrophy, and shedding of renal tubular epithelial cells in mouse kidneys [97].

For the liver, nanoparticles can reach and accumulate within it via the circulatory system. Observations from in vitro experiments using mouse (AML-12) and human (L02) liver cell lines show that following exposure to MNPs, the NF-κB pathway becomes activated, leading to a rapid increase in serum inflammatory markers TNF-α and IL-6. Concurrently, elevated levels of alanine aminotransferase (ALT) and aspartate aminotransferase (AST) are observed, indicating pronounced hepatitis symptoms [27]. Similarly, a chronic exposure study of PS-MNPs in mice showed that particles of 80 nm and 0.5 μm significantly induced hepatic inflammatory responses, as reflected by elevated protein expression levels of IL-1β and IL-6, accompanied by a marked upregulation of the macrophage marker CD 68 [98]. In addition, the study found that PS-MNPs caused hepatic lipid deposition. They disrupted lipid metabolism by interfering with pathways such as fatty acid elongation and unsaturated fatty acid synthesis, and significantly upregulated the mRNA expression levels of genes associated with fatty acid oxidation, synthesis, and transport [98]. Collectively, MNPs not only directly trigger hepatic inflammatory responses but also synergistically exacerbate liver tissue injury by disturbing lipid metabolism and promoting immune cell infiltration.

Additionally, lipopolysaccharide (LPS) secreted during intestinal flora dysbiosis may activate Toll-like receptor (TLR)-4. This further induces the Nlrp3 inflammasome to synthesize IL-1β and IL-18, triggering inflammation that subsequently affects the bone marrow and impairs hematopoiesis as well as the trafficking of HSPCs [99]. Studies have found that elevated levels of Actinobacteria and Proteobacteria in the gut microbiota can lead to a differentiation bias in LSK cells, skewing them toward common myeloid progenitors (CMPs) that produce erythrocytes, while the population of common lymphoid progenitors (CLPs) responsible for generating immune cells gradually decreases, resulting in anemia [100]. Furthermore, under homeostatic conditions, the short-chain fatty acid (SCFA) butyrate, produced by the gut microbiota, promotes macrophage-mediated erythrophagocytosis. This process supplies iron for erythropoiesis. When gut microbiota dysbiosis occurs, it impairs erythrocyte phagocytosis by modulating macrophage function via SCFA, thereby disrupting iron metabolism through the microbiota-macrophage-iron axis and impairing hematopoietic function [101]. Existing research has demonstrated that exposure to MNPs can induce gut microbiota dysbiosis. For instance, in mice exposed to PS-MNPs, the abundance of Proteobacteria and Actinobacteria in the gut microbiota increased, accompanied by disturbances in neurotransmitter metabolism [102,103,104]. These findings raise the possibility that long-term MNP accumulation could contribute to hematopoietic dysfunction via gut microbiota dysbiosis, although this proposed pathway remains entirely hypothetical and has not been directly demonstrated in any single experimental model.

**Table 4:** Possible toxicity mechanisms of MNPs accumulation in hematopoietic-related organs.

Hematopoietic-Related Organs	Type of Evidence Source (Study Design and (Species))	Pathways	Micro-Level Changes	Macro-Level Changes	References
Kidneys	In vivo design (Rat/Mouse)	Nrf2 pathway (inhibition)	MDA: ↑ *	Lymphocytic infiltration	[95,96,97]
			Antioxidant enzyme activity: ↓		
				Mild glomerular atrophy	
		Ferroptosis/Mitochondria-Related Pathway (activation)	Renal inflammation-associated factors: ↑		
			Ferritin autophagy-related genes and proteins: ↑		
				Shedding of renal tubular epithelial cells	
			Apoptosis-related genes: ↑		
Liver	In vitro design (human/mouse)/In vivo design (Mouse)	NF-κB pathway (activation)	TNF-α, IL-6, IL-1β: ↑	Hepatitis	[27,98]
			CD68: ↑	Liver fibrosis	
		Pathways related to lipid metabolism and fatty acid metabolism (activation)			
			mRNA related to fatty acid oxidation, synthesis and transport: ↑	Impaired liver metabolism and function	
Bone marrow microenvironment	In vivo design (Mouse)	TLR4-Nlrp3 inflammasome pathway (activation)	LPS: ↑	Gut microbiota imbalance	[102,103,104]
				Iron metabolism disorder	
			IL-1β, IL-18: ↑	Anemia	
		SCFA-Macrophage-Iron Axis (disruption)		Neurotransmitter Metabolic Disorder	
			Actinomycetes, Proteobacteria: ↑	LSK cell differentiation bias (CMP ↑, CLP ↓)	
				Hematopoietic dysfunction	

* “↑” indicates an increase in the level of expressed of this indicator; while “↓” indicates a decrease in the level of expression of this indicator. Nrf2: Nuclear factor erythroid 2-related factor 2; MDA: Malondialdehyde; NF-κB: Nuclear factor kappa-B; TNF-α: Tumor necrosis factor-α; IL-6: Interleukin-6; IL-1β: Interleukin-1β; CD68: Cluster of differentiation 68; TLR4: Toll-like receptor 4; Nlrp3: NOD-like receptor pyrin domain-containing 3; LPS: Lipopolysaccharide; IL-18: Interleukin-18; SCFA: Short-chain fatty acid; LSK: Lin^−^Sca-1^+^c-Kit^+^; CMP: Common myeloid progenitor; CLP: common lymphoid progenitors.

## 6. Exploratory Experimental Avenues for Addressing Micro- and Nanoplastic Exposure and Accumulation

### 6.1. Advances in Methods for Reducing Micro- and Nanoplastics in the Environment

With the advancement of urbanization and industrialization, the demand for plastics continues to grow. Particularly since the onset of the COVID-19 pandemic, increased public demand for medical products such as masks may have contributed to the continuous accumulation of MNPs in the environment [105]. Numerous methods are currently available to address this issue, among which biodegradation is one of the most recognized and environmentally friendly technologies. Studies have found that native microorganisms such as bacteria or fungi can be isolated from plastic dumping sites or wastewater. With the aid of enzymes they secrete, these organisms can destabilize the charges on plastics, thereby promoting the breakdown of NPs [106]. In the future, these microorganisms hold promise as important agents for tackling plastic pollution in both terrestrial and aquatic ecosystems. Particularly noteworthy are microalgae, which utilize sunlight as their primary energy source and display considerable biosorptive and biodegradative capabilities for removing MNPs from aquatic environments. The diverse functional groups on microalgal cell walls enable effective adsorption and immobilization of MNPs, while their intracellular metabolic activity and secreted enzymes further contribute to the biodegradation and breakdown of plastic polymers. These features collectively support the ability of microalgae to sequester and transform MNPs in contaminated water systems. Although quantitative data on removal efficiency, scalability, and long-term stability remain limited in current research, the unique advantages of photosynthetic microorganisms highlight their promising potential as sustainable and eco-friendly candidates for advancing MNPs remediation strategies in natural environments [107].

### 6.2. Exploration of Micro- and Nanoplastic Toxicity Mitigation from an “Medicinal and Edible Homology” Perspective

Medicinal and edible homology (MEH) refers to natural resources that possess both edible and medicinal value, embodying the core philosophy that medicine originates from daily food, food offers therapeutic effects, and medicinal ingredients can be naturally integrated into dietary habits [108,109]. With growing public health awareness, research and product development related to MEH have attracted increasing attention [110]. In this context, we have compiled and summarized existing studies regarding natural substances aligned with the connotation of MEH, focusing on their potential to alleviate damage induced by MNPs.

Research has demonstrated that the anthocyanin cyanidin-3-O-glucoside (C3G), extracted from bayberry, can reduce the intracellular accumulation of MNPs in human colon Caco-2 cells by increasing ATP levels, which in turn enhances the activity of the intracellular ABC transporter [111]. Additionally, studies in mice have also shown that this food-derived substance exerts a protective effect against PS-induced toxicity and gut microbiota dysregulation [111,112]. As for resveratrol, which is widely present in foods such as grapes and peanuts, animal experiments have demonstrated that administration of 50 mg/L resveratrol to SD rats following MNP exposure significantly alleviated the MNP-induced increase in creatinine levels. Through the modulation of gut microbiota and its metabolites, resveratrol treatment effectively ameliorated the elevated blood pressure and renal oxidative stress caused by high-dose MNP exposure [113]. It should be emphasized that all of the above findings are derived from in vitro or animal studies employing MNP concentrations substantially higher than estimated human environmental exposure. Whether these bioactive compounds exert comparable protective effects at physiologically relevant doses in humans has not been investigated. Before any translational conclusions can be drawn, well-designed dose–response studies using environmentally relevant MNP concentrations are needed, followed by validation in human-derived models or clinical settings. At present, these findings identify potential mechanistic leads for future research into dietary approaches to MNP-related toxicity, but they do not constitute evidence sufficient to support specific dietary recommendations.

Furthermore, it has also been reported that supplementation with probiotics (4.2 g/(kg·bw) bifico) can reverse the NP-induced reduction in the weight of hematopoietic-related organs including the spleen and thymus in a mouse model. Probiotic supplementation also significantly restored indicators associated with bone marrow hematopoietic stem cell renewal, suggesting an exerting a protective effect against MPs-induced toxicity in mice [114].

Since the aforementioned findings are all based on in vitro experiments or animal experiments, further investigation is required to determine the effective concentrations of these substances for alleviating MNP-induced damage in humans, and whether similar effects can be observed in the human body remains to be confirmed by future studies. Moreover, the concentration of MNPs encountered in daily life is likely much lower than that used in experimental settings, making their effects on the human body more consistent with experimental models of low-dose long-term exposure. The direct effects of MNPs on the human body may be difficult to quantify, thus affecting the assessment of the therapeutic benefits of dietary therapy. Nevertheless, as these substances are derived from readily accessible foods and are considered safe for most healthy individuals, the proposed strategies still hold promise as potential dietary measures for preventing MNPs-induced health damage.

## 7. Conclusions

As an emerging contaminant, MNPs have attracted widespread attention. However, direct evidence regarding the effects of MNPs on the human hematopoietic system is extremely scarce, with the majority of current knowledge derived from animal models and in vitro studies, and the underlying mechanisms are not yet fully understood. This narrative review summarizes the accumulation patterns of MNPs in various tissues and organs as observed predominantly in preclinical models, along with their underlying influencing factors. It provides a detailed account of the potential toxic effects of MNPs, particularly their possible toxicity within the hematopoietic system, and discusses the molecular mechanisms proposed to date. Preclinical evidence suggests that MNPs may contribute to hematotoxicity through inflammatory responses and metabolic disturbances, and may inhibit the differentiation of bone marrow hematopoietic stem cells into granulocytes and megakaryocytes, thereby reducing peripheral blood cell counts, although these mechanisms have not been confirmed at environmentally relevant doses or in human systems. Preliminary transcriptomic evidence from a single mouse study also suggests that MNPs may disrupt normal hematopoietic function via the JAK/STAT pathway, though this requires independent replication. Based on these findings, we highlight emerging but preliminary research into environmental biodegradation approaches, including the use of microorganisms such as microalgae, and early-stage investigations into dietary bioactive compounds inspired by the concept of medicinal and edible homology, as areas that may warrant further investigation.

Given that the conclusions of this review rest almost entirely on animal and in vitro evidence, the translational value of existing findings is considerably constrained. To address this gap, we propose the following two research directions. On one hand, epidemiological studies utilizing population-based cohorts are encouraged to longitudinally assess the actual impact of MNPs on human health. On the other hand, in basic research, the establishment of three-dimensional human cell culture systems or organoid models is recommended to better recapitulate the human physiological environment, thereby enhancing the translational relevance of research findings to human populations.

## Figures and Tables

**Table 1:** Material Types of MNPs Present in Air and Diet.

Designation	Abbreviation	Primary Method of Intake	Applications in Daily Life
Polystyrene	PS	Respiration/diet	Stationery clips, children’s building blocks, television casings, disposable lunch boxes, yogurt pots, etc.
Polyethylene	PE	Respiration/diet	Plastic buckets, domestic water supply pipes, food packaging film, plastic bottles, etc.
Polypropylene	PP	Respiration/diet	Plastic basins, plastic chairs, storage crates, water cups, etc.
Polyamide	PA	Respiration/diet	Comb, toothbrush, mesh bag rope, Food packaging, tableware, etc.
Polyethylene Terephthalate	PET	Respiration/diet	Cosmetic packaging, beverage bottles, soy sauce bottles, etc.
Polyesteramide	PEA	diet	Beverage bottles, food packaging, etc.
Polyvinyl Chloride	PVC	Respiration/diet	Window and door profiles, drainage pipes, artificial leather, household plastic film, etc.
Expanded Polystyrene	EPS	diet	Fresh-keeping transport packaging for fruit and seafood, insulation materials for cold storage facilities, etc.
Polyethylene Succinate Terephthalate	PEST	Respiration	Laminating materials in clothing and footwear production
Low-Density Polyethylene	LDPE	diet	Food packaging film, agricultural film, bottle caps, etc.
High-Density Polyethylene	HDPE	diet	Beverage bottles, food storage containers, cooking oil bottles, etc.

**Table 3:** Deposition mechanisms and associated alterations of MNPs in blood cells and hematopoietic-related organs.

Classification	Primary Depositing Cells/Organs	Subcategory	Deposition Pattern	Changes	Type of Evidence Source (Study Design and (Species))	References
Blood cells	Leukocytes	Neutrophils	Interactions of cell membranes	Inflammatory response	ES ^1^: in vitro design (Artificial model/Rat) EC ^2^: in vitro design (Human/Artificial model/Rat)	[36,37,38,58,59,60]
			Clathrin- and caveolin-mediated endocytosis	Increase in quantity		
			Phagocytose vascular endothelial cells	Dysfunction of neutrophil extracellular traps (NETs)	ES: in vitro design (Human/Artificial model/Rat) EC: in vivo and vitro design (Mouse)	
		Macrophage	Phagocytosis	Lysosomal damage	ES: in vitro design (Artificial model/Rat) EC: in vitro design (Rat/Artificial model/zebrafish)	[36,61,62,63,64,65,66,67,68]
				Autophagy inhibition		
				Cell death		
	Thrombocytes	-	Charge adsorption	Abnormal aggregation	ES: in vitro design (Human) EC: in vitro design (Human)/in vivo design (Mouse)	[40,43,44,69,70,71,72]
			Activation of the classical coagulation pathway			
			Activation of the JAK1/STAT3/TF signaling pathway		ES: in vivo design (Mouse) EC: in vitro design (Human)/in vivo design (Mouse)	
Hematopoietic-related organs	Liver (before birth)	-	Blood circulation	Hepatic steatosis and fibrosis	ES: in vivo design (mouse) EC: in vitro design (Human)	[9,22,73,74,75,76]
			Gut-liver axis			
	Kidneys	-	Blood circulation	Reduced glycolytic activity	ES: in vivo design (mouse) EC: in vitro design (Human)	[9,19,57,77,78,79,80,81,82]
				Energy metabolism disorder		
			Filtration and reabsorption	Disorders of lipid metabolism		
				Renal impairment		

^1^ ES: Evidence source of sedimentary pattern; ^2^ EC: Evidence source of change.

## Data Availability

No new data were created or analyzed in this study. Data sharing is not applicable to this article.
